# Hearing Loss and Middle Ear Effusion in Nasopharyngeal Carcinoma Following Radiotherapy: Dose–Response Relationship and Normal Tissue Complication Probability Modeling *

**DOI:** 10.1055/s-0045-1805045

**Published:** 2025-07-03

**Authors:** Prem Wungcharoen, Anussara Prayongrat, Napadon Tangjaturonrasme

**Affiliations:** 1 Department of Otolaryngology, Faculty of Medicine, Chulalongkorn University, Bangkok, Thailand; 2 Division of Radiation Oncology, Department of Radiology, Faculty of Medicine, Chulalongkorn University and King Chulalongkorn Memorial Hospital, Bangkok, Thailand; 3 Division of Head and Neck Surgery, Department of Otolaryngology, Faculty of Medicine, Chulalongkorn University, Bangkok, Thailand

**Keywords:** nasopharyngeal neoplasms, hearing loss, radiotherapy, proton, otitis media with effusion

## Abstract

**Introduction** Radiotherapy is the primary treatment for nasopharyngeal carcinoma. Radiation exposure to the cochlea and middle ear can cause hearing loss.

**Objective** To develop a multivariable normal tissue complication probability (NTCP) model to predict the risk of hearing impairment in nasopharyngeal cancer patients based on clinical and radiation dosimetry features and to identify the key factors associated with hearing loss.

**Methods** A retrospective review of 229 patients was conducted. We recorded the audiometry and presence of middle ear effusion (MEE) and compared findings before and after therapy. The factors included age, gender, signs and symptoms at presentation, tumor staging, prescribed dose at the tumor and high-risk nodal region, cochlea, and concurrent chemotherapy treatment. The model was formulated using multivariate logistic regression.

**Results** Age of more than 50 years, high primary staging, and dose at the cochlea > 43 Gy were major risk factors for sensorineural hearing loss. The final NTCP model for hearing loss comprised age and cochlea dose with an area under the curve (AUC) of 0.644; the predicted risk ranged from 15.84 to 44.52%. Locally advanced disease and cochlea dose greater than 44 Gy were risk factors for MEE; the predicted risk ranged from 20.42 to 51.99%.

**Conclusion** Age over 50, T stages 3 and 4, and > 43 Gy dose to the cochlea were significantly associated with an increased risk of sensorineural hearing loss and MEE. The developed NTCP model provides information to predict these risks, aiding in treatment planning and decision-making to avoid complications.

## Introduction

Nasopharyngeal carcinoma (NPC) is common in the Asian population, particularly in the southeast region, including Thailand. According to the Global Cancer Observatory (GLOBOCAN) 2020, there were 2,316 new NPC cases and 1,482 deaths, with an annual incidence of 2.6/100 thousand for men and 1/100 thousand for women, in 2020. 1 2 According to the National Comprehensive Cancer Network guideline, the current gold standard for treatment is radiation with or without chemotherapy. 3 Intensity-modulated radiotherapy (IMRT) and volumetric modulated arc therapy (VMAT) techniques are considered as the standard treatment for NPC. 4 5 6 A type of photon or X-ray beam therapy, IMRT has gained popularity because of its ability to deliver radiation more precisely to the tumor while sparing the nearby radiosensitive tissues, such as those of the brain, orbits, cochlea, and spinal cord. 7 Volumetric modulated arc therapy is another IMRT technique, in which the machine rotates around the patient delivering radiation beams in an arc-like pattern. The radiation dose intensity was determined by the amount of radiation left after passing through tissues, type of radiation particles, volume of the irradiated organ, and type of organ. 8 Patient factors, such as underlying disease, smoking behavior, and concurrent chemotherapy treatment, all had considerable impact. 9 Proton beam therapy is a novel technique that uses the proton particle for energy delivery. Current research supports the use of proton beam therapy since it is associated with less side effects when compared with photon beam therapy. 8 10 However, due to its cost and limited availability, its usage should be reserved for individuals who are expected to experience significant side effects from regular IMRT or VMAT.

Despite modern techniques, radiotherapy (RT) can cause adverse effects on surrounding tissues, such as hypothyroidism, nasopharyngeal fibrosis, chronic rhinosinusitis, and hearing loss. 11 12 Approximately 73% of patients who underwent RT to the head and neck developed conductive, sensorineural, or mixed hearing loss. 13 Conductive hearing loss commonly manifests at 3 months after treatment due to middle ear effusion (MEE) (8–29%), tympanic membrane stiffness (15–32%), and ossicular fibrosis (5%), whereas sensorineural hearing loss develops later due to cochlea or auditory nerve injury. 14 The relationship between radiation dose to auditory structures and toxicity has been previously reported. 15 16 A mathematical model to predict the risk of radiation-induced ototoxicity in NPC patients was mostly based on radiation dose to the cochlea, but did not consider other clinical factors. 17 18 19 However, the risk factors of ototoxicity in NPC patients include radiation to the auditory pathway (internal acoustic canal, middle ear, cochlea, and Eustachian tube) and the use of cisplatin chemotherapy based on several studies in the non-IMRT 17 18 20 21 and IMRT eras. 22 23 Patients aged 50 years and older often experience a higher incidence of age-related hearing loss, which can exacerbate the impact of radiation-induced hearing impairment, making these findings more prominent and troublesome in this population. 24

Therefore, the purpose of the current study is to develop a multivariable normal tissue complication probability (NTCP) model that can predict the risk of hearing impairment using clinical and radiation dosimetry features. We also aimed to estimate the likelihood of audiologic complications following RT, to identify the factors that cause hearing loss in NPC patients who have undergone IMRT or VMAT, and to report the pattern and severity of hearing loss in NPC patients following RT.

## Methods

Through a chart review, this retrospective cohort, prognostic study included patients: 1) whose NPC diagnosis was confirmed by histopathology and who were treated with IMRT or VMAT at a dose of 66 to 70 Gy in 30 to 35 fractions, with or without chemotherapy, at King Chulalongkorn Memorial Hospital (KCMH) between December 2008 and December 2019; 2) with available radiotherapy dose–volume histogram (DVH) data; and 3) with the audiogram and computed tomography (CT) scan reported prior to and following treatment. We also excluded patients: 1) who previously received radiation or chemotherapy in the head and neck region; 2) with incomplete prescribed treatment plan; 3) with insufficient information to identify the relevant factors; for instance, the middle ear was not visible on the CT scan, image artifacts, or no audiogram report; and 4) who previously had MEE prior to treatment.

Data was collected from the electronic medical records of the NPC patients who received treatment at the KCMH otolaryngology and radiation oncology department. The demographic data included age at the initial RT administration, sex, Tumor, Node, and Metastasis (TNM) staging, 3 chemotherapy (in line with the National Comprehensive Cancer Network (NCCN) guidelines, 3 the chemotherapy regimens in this study were all cisplatin-based) and dose-volume histogram (DVH) data (mean and maximum radiation dose [Gy] corresponding to the following volumes under the supervision of an RT specialist: [1] gross tumor volume [GTV], [2] clinical target volume [CTV], [3] planning target volume [PTV], and [4] each cochlea). Posttreatment audiogram and CT scan were collected at least 3 months following the last treatment session.

### Treatment

All patients were immobilized in the supine position with a tailored head–shoulder thermoplastic mask, then a CT simulation was performed. Magnetic resonance simulation was performed on every patient and co-registration with the CT images. Two or three PTVs were designated as follows: PTV-high risk (PTV-HR) was defined as gross tumor and gross pathologic lymph nodes (LNs) and received doses of 70 Gy; PTV-intermediate risk was defined as the subclinical disease and received prophylactic doses of 60 to 70 Gy; and PTV-low risk (PTV-LR) was defined as the elective LN region (bilateral cervical LN levels II–V, VII) and received doses of 50 to 56 Gy. A simultaneous integrated boost (SIB) or sequential cone-down boost of 20 Gy to the PTV-HR was selected by the physician's decision. IMRT or VMAT was applied. Radiotherapy planning was performed using the Eclipse treatment planning system (Varian Medical Systems Inc., Palo Alto, CA, USA) version 6.5–15.0. Radiation was delivered using a linear accelerator (Varian Medical System Inc.) Treatment verification was performed regularly with daily electronic portal images and weekly cone-beam CT.

All patients received concurrent chemoradiotherapy, with or without neoadjuvant or adjuvant chemotherapy. Cisplatin was administered weekly or tri-weekly concurrently with definitive radiotherapy at a dose of 70 Gy in 33 to 35 fractions. Neoadjuvant or adjuvant chemotherapy regimens, including platinum-based (cisplatin/carboplatin), infusion fluorouracil (5 FU), paclitaxel, or gemcitabine, were given at 3- or 4-week intervals for 3 cycles.

### Outcome Measurements

The primary outcome of the present study was the incidence of hearing loss, which was determined by using an audiogram. Pure tone average (PTA) of both air and bone conductions (3 frequency protocols) as well as the duration of the threshold worsening by > 10 dB HL were collected as a secondary outcome of the study. Middle ear effusion was determined by the presence of fluid between −5 and 20 Hounsfield units (HUs) in the middle ear cavity on the CT scan.

### Statistical Analysis

Categorical data were analyzed using the Chi-squared or Fisher's exact test. Continuous data were analyzed using the Wilcoxon signed rank-sum test or Student t-test. The significant factors for toxicity were identified using binary logistic regression with significance set at a *p* -value of < 0.05. Multivariate logistic regression (forward stepwise selection/bootstrapping) was used to create an NTCP model:



with S(x) = β _0_ + β _1_ x _1_ + β _2_ x _2_ + … + β _n_ x _n_ ,

in which β _0_ is a constant and β _1_ , …, β _n_ are the logistic regression coefficients of the variables x _1_ , x _2_ , …, x _n_ , respectively.

The performance of the model was assessed by the area under the receiver operating characteristic curve (AUC) analysis and the Hosmer-Lemeshow goodness-of-fit test, whereas a non-significant *p* -value of > 0.05 indicated good predictive ability. Finally, internal validation was performed with the 10-fold cross validation. The data were analyzed using STATA (StataCorp LLC, College Station, TX, USA), version 15.1.

The present study was approved by the Institutional Review Board of the Faculty of Medicine at Chulalongkorn University (COA No. 303/63). The need to obtain patient informed consent was waived by the institutional review board because of the retrospective nature of the study.

## Results

**Fig. 1:**
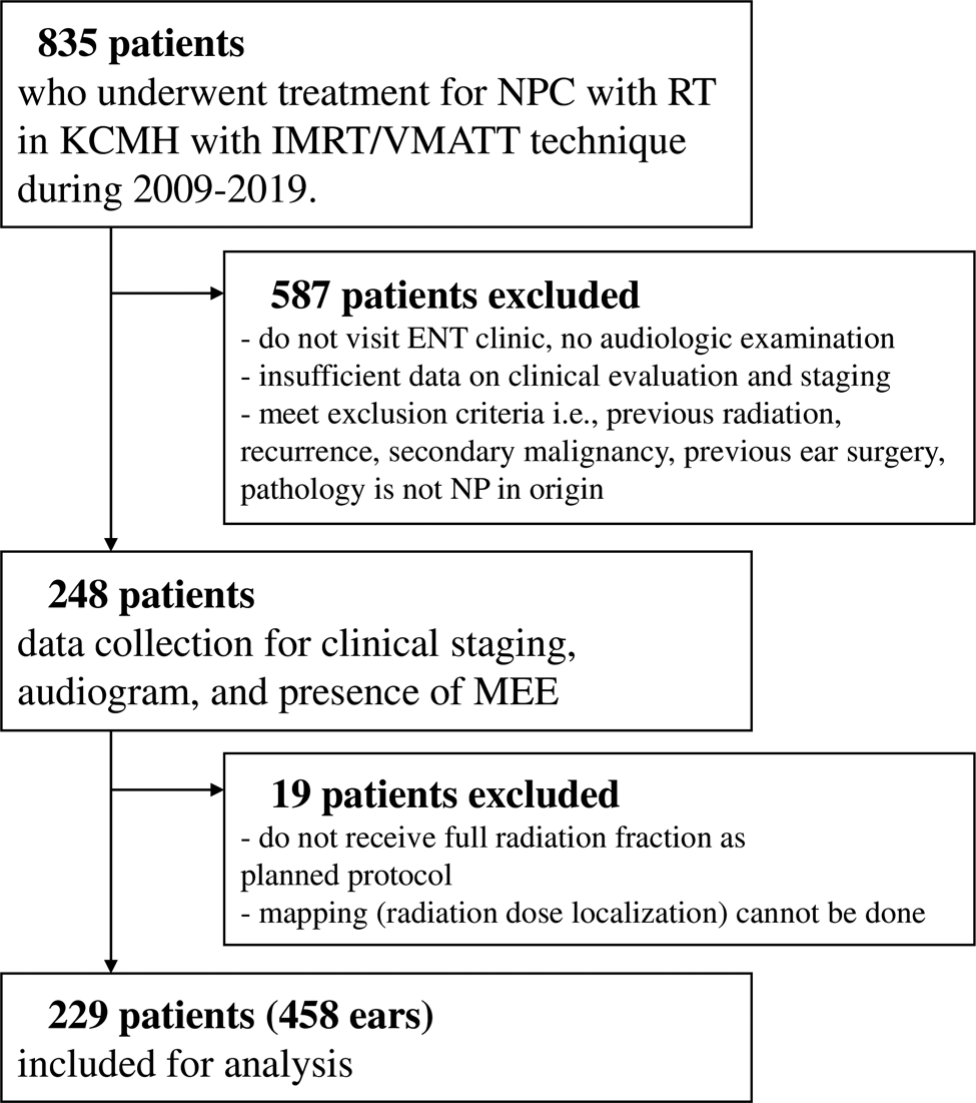
Flow diagram of patient recruitment.

Altogether, 835 NPC patients receiving photon-based IMRT were identified from the database, but 587 cases were removed based on the exclusion criteria. The data of the remaining 248 patients were reviewed. Additionally, 19 patients were further excluded because they were not given full radiation treatment as planned. Finally, 229 participants with a total of 458 ears were included in the analysis, as demonstrated in Fig. 1 . The demographic data, TNM classification, and treatment characteristics are shown in Table 1 . Most patients were men. The patients' mean age was 49.43 years (standard deviation [SD] = 14.03). Nasal symptoms were the most common presentation (53%) followed by neck mass (45%), auditory symptoms (44%), such as hearing loss and aural fullness. The other signs and symptoms were neural involvement (11%), such as diplopia, facial numbness, or headache. The maximum dose (D _max_ ) to GTV was 74.25 Gy (SD 2.48); CTV, 72.61 Gy (SD 2.84); PTV-70, 71.48 Gy (SD 3.22); and CV, 45.45 Gy (SD 13.19). The median duration of hearing follow-up after therapy was 130 days. Most patients received VMAT rather than the conventional IMRT and were concurrently submitted to chemotherapy during RT (93.01%).

**Table 1:** Demographic data according to the patients' baseline and treatment characteristics

Baseline characteristics at presentation		Patients ( *n* = 229)	Percentage (%)	Treatment characteristics		Patients ( *n* = 229)	Percentage (%)
Gender	Female	81	35.37	Radiation technique	IMRT	24	10.49
	Male	148	64.63		VMAT	205	89.51
Age	< 50 years	102	44.54	Concurrent chemotherapy	No	16	6.99
	≥ 50 years	127	55.46		Yes	213	93.01
Signs and symptoms	Nasal	122	53.27	Follow-up audiogram (days)	< 31	3	1.31
	Aural	102	44.54		31–90	74	32.31
	Neck mass	103	44.97		91–180	55	24.02
	Neural	26	11.35		181–365	48	20.97
TNM stage	T (%)	N (%)	M (%)		> 365	49	21.39
0		50 (21.9)	222 (96.9)	Average follow-up (days)		254.86	
1	66 (28.8)	61 (26.6)	7 (3.1)	Median follow-up (days)		130	
2	58 (25.3)	92 (40.2)					
3	55 (24.0)	26 (11.4)					
4	50 (21.8)						

Abbreviations: IMRT, intensity-modulated radiotherapy; M, metastasis; N, node; T, tumor; TNM, tumor, node, and metastasis staging; VMAT, volumetric modulated arc therapy.

### Sensorineural Hearing Loss

Hearing loss was observed in 96 out of 229 (41.92%) patients. The demographic data were divided into 2 groups based on post RT hearing status as presented in Table 2 . There was a statistically significant difference in age, T stage, and mean radiation exposure (D _mean_ ) to the cochlea of > 43 Gy between the two groups. The cochlea dosage threshold, or cut-off value, of 43 Gy was calculated using the maximum AUC of 0.663. The results of the univariate and multivariate logistic regression analyses are depicted in Table 3 . In the univariate analysis, age > 50 years, stages 3 and 4, and cochlea dose > 43 Gy were related to odds ratios (ORs) of 1.68, 1.60, and 2.58, respectively. The final NTCP model comprised age and cochlea dose with an AUC of 0.644 (precision of 70.1%). The calculated risk of hearing loss ranged between 15.84 and 44.52%, as illustrated in Table 4 .

**Table 2:** Comparison between the normal hearing and hearing loss groups

Analyzed factors	Group I: normal hearing ( *N* = 133)	Group II: hearing loss ( *N* = 96)	*p* -value
Gender: male – n (%)	85 (63.9)	63 (65.6)	0.79
Age in years (IQR)	49 (39–56)	55.5 (43.5–61)	0.002*
Age groups: n (%)			
• < 50 years • ≥ 50 years	68 (51.1) 65 (48.9)	34 (35.4) 62 (64.6)	0.04*
Symptoms at presentation: n (%)			
Aural	55 (41.4)	47 (49)	0.25
Nasal	66 (49.6)	56 (58.3)	0.19
Mass	59 (44.4)	44 (45.8)	0.83
Neural	11 (8.3)	15 (15.6)	0.11
Staging: n (%)			
T3–4	53 (39.6)	52 (54.2)	0.03*
N2–3	64 (47.8)	54 (56.3)	0.20
M1	2 (1.5)	5 (5.2)	0.11
Treatment protocol			
D _max_ : GTV-NP (IQR) in cGy	7,428 (7,301–7,529)	7,458 (7,306–7,578)	0.42
D _max_ : CTV-NP (IQR) in cGy	7,274 (7,156–7,431)	7,302 (7,107.5–7,455.5)	0.53
D _max_ : PTV-70 (IQR) in cGy	7,192 (6,997–7,325)	7,252 (6,990–7,376.5)	0.32
Using chemotherapy: n (%)	119 (89.5)	94 (97.9)	0.28
**Analyzed factors**	**Group I: post-RT normal hearing, ears (** ***N*** ** = 321)**	**Group II: post-RT hearing loss, ears (** ***N*** ** = 137)**	***p*** **-value**
D _mean_ : cochlear dose (IQR) in cGy	4,157 (3,588–5,017)	4,817 (3,893–5,810)	< 0.001*

Abbreviations: CTV, clinical target volume; D _max_ , maximum dose; GTV, gross tumor volume; IQR, interquartile range; M, metastasis; N, node; NP, nasopharynx; RT, radiotherapy; T, tumor.

Note: Bold values represent *p* -value < 0.05.

**Table 3:** Univariate and multivariate analyses of hearing loss

Analyzed factors		Univariate analysis		Multivariate analysis	
Factors	Reference	OR (95%CI)	*p* -value	aOR (95%CI)	*p* -value
Male	Female	1.12 (0.73–1.71)	0.06		
Age group ≥ 50 years	< 50	1.68 (1.11–2.53)	0.01*	1.67 (1.09–2.55)	0.02*
T 3–4	0–2	1.60 (1.07–2.39)	0.02*		
N 2–3	0–1	1.42 (0.95–2.13)	0.29		
M1	0	1.79 (0.61–5.27)	0.29		
Cochlear dose≥ 4,300 cGy	< 4,300	2.58 (2.69–3.92)	< 0.001*	2.57 (1.68–3.92)	< 0.001*

**Abbreviations:** 95%CI, 95% confidence interval; aOR, adjusted odds ratio; OR, odds ratio; M, metastasis; N, node; T, tumor.

**Table 4:** Normal tissue complication probability model and calculated risk factor for hearing loss

Age at RT ≥ 50 years	Cochlear dose > 43 Gy	Modifying factor S (x)	Risk factor for hearing loss (%)
Yes	Yes	−0.22	44.52
Yes	No	−1.16	23.86
No	Yes	−0.73	32.51
No	No	−1.67	15.84

Abbreviation: RT, radiotherapy.

**Fig. 2:**
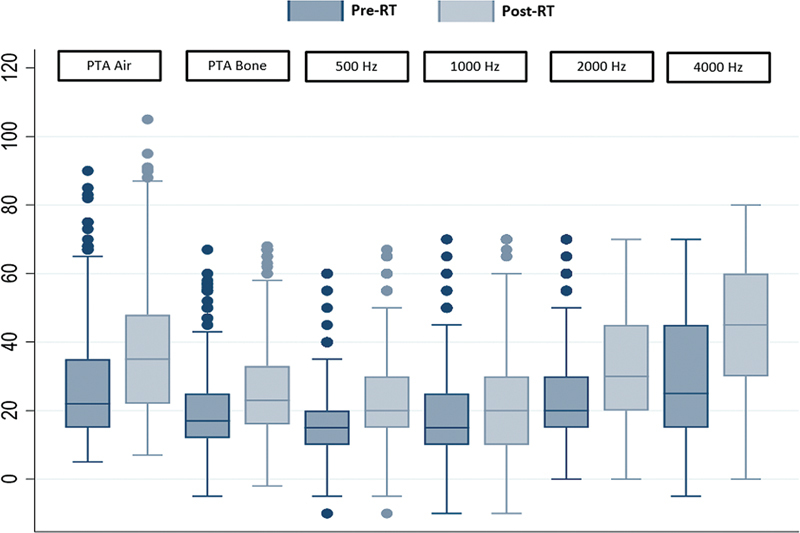
Boxplot diagrams of audiograms (dB HL) between pure-tone audiometry and specific frequencies of bone conduction.

Fig. 2 depicts the pre- and post-treatment audiologic characteristics of air conduction pure tone audiometry (PTA), bone conduction PTA, and bone conduction specific frequencies. Pure tone audiometry for air and bone conductions decreased by 8.77(± 14.93) and 7.7(± 11.82) dB after RT, respectively. The hearing loss was detected in every frequency and was more pronounced at high frequencies.

### MEE

Middle ear effusion had been newly diagnosed in 92 patients (42.79%) out of the 215 patients. The demographic data for groups with and without MEE are presented in Table 5 . There was a statistically significant difference between the 2 groups in terms of presenting symptoms (aural and neural), T-stage, and radiation exposure to cochlea. The cochlea dosage threshold, or cut-off value, of 44 Gy was calculated using the maximum AUC of 0.659. The results of the uni and multivariate logistic regression analyses are depicted in Table 6 . In the univariate analysis, T-stages 3 and 4 and mean radiation exposure (D _mean_ ) > 44 Gy showed ORs of 2.95 and 1.80, respectively. The final NTCP model was established utilizing T-stage and cochlea dose with an AUC of 0.658 (precision of 66.1%). The calculated risk of developing MEE ranged between 20.42 and 51.99%, as illustrated in Table 7 .

**Table 5:** Comparison between the no MEE and the new MEE groups

Analyzed factors	Group I: no MEE post-RT ( *N* = 123)	Group II: new MEE post-RT ( *N* = 92)	*p* -value
Gender: male – n (%)	77 (62.6)	61 (66.3)	0.58
Age in years (IQR)	50 (40–59)	52.5 (41.5–58)	0.55
Age groups: n (%)			
• < 50 years • ≥ 50 years	49 (39.8) 74 (60.2)	50 (54.3) 42 (45.7)	0.72
Symptoms at presentation: n (%)			
Aural	46 (37.4)	47 (51.1)	0.04*
Nasal	67 (54.5)	48 (52.2)	0.74
Mass	51 (41.5)	48 (52.2)	0.12
Neural	8 (6.5)	16 (17.4)	0.01*
Staging: n (%)			
T3–4	45 (36.6)	53 (57.6)	0.002*
N2–3	62 (50.4)	49 (53.3)	0.68
M1	6 (4.9)	1 (1.1)	0.12
Treatment protocol			
D _max_ : GTV-NP (IQR) in cGy	7,458 (7,294–7,540)	7,411.5 (7,302.5–7,577.5)	0.92
D _max_ : CTV-NP (IQR) in cGy	7,272 (7,143–7,438)	7,293.5 (7,165.5–7,453.5)	0.45
D _max_ : PTV-70 (IQR) in cGy	7,190 (6,982–7,357)	7,234 (7,007.5–7,341)	0.42
Using chemotherapy, n (%)	113 (91.9)	89 (96.7)	0.55
**Analyzed factors**	**Group I: no MEE post RT, ears (** ***N*** ** = 123)**	**Group II: new MEE post RT, ear (** ***N*** ** = 92)**	***p*** **-value**
D _mean_ : cochlear dose (IQR) in cGy	4,255 (3,591–5,314)	4,601 (3,765–5,687)	< 0.001*

Abbreviations: CTV, clinical target volume; GTV, gross tumor volume; IQR, interquartile range; MEE, middle ear effusion; M, metastasis; N, node; NP, nasopharynx; PTV-70, planning target volume receiving 70 Gy; RT, radiotherapy; T, tumor.

Note: Bold values represent *p* -value < 0.05.

**Table 6:** Uniand multivariate analyses of middle ear effusion

Analyzed factors		Univariate		Multivariate	
Factors	Reference	OR (95%CI)	*p* -value	aOR (95%CI)	*p* -value
Male	Female	1.05 (0.67–1.67)	0.80		
Age group ≥ 50 years	< 50	1.09 (0.71–1.69)	0.69		
T3–4	0–2	2.95 (1.88–4.63)	< 0.001*	2.76 (1.75–4.35)	< 0.001*
N2–3	0–1	1.04 (0.67–1.61)	0.86		
M1	0	0.21 (0.03–1.66)	0.14		
Cochlear dose (cGy)	< 4,400	1.80 (1.16–2.81)	0.01*	1.54 (0.98–2.43)	0.06

**Abbreviations** : 95%CI, 95% confidence interval; aOR, adjusted odds ratio; OR, odds ratio; M, metastasis; N, node; T, tumor.

S(x) = −1.36 + [1.01“if T stage 3-4] + [0.43 “if cochlear dose ≥ 4,400”]

**Table 7:** Normal tissue complication probability model and calculated risk factor for middle ear effusion

T-stages 3 and 4	Cochlear dose > 44 Gy	Modifying factor S (x)	Risk factor for MEE (%)
Yes	Yes	0.08	51.99
Yes	No	−0.35	41.33
No	Yes	−0.93	28.29
No	No	−1.36	20.42

**Abbreviation** : MEE, middle ear effusion; T, tumor.

## Discussion

It is acknowledged that hearing loss is a common adverse effect following head and neck RT, particularly when the inner ear is included in the irradiated field and the radiation dose is high. 25 Hearing loss is progressive and irreversible, 14 often occurring at least 3 months after the last treatment. Typically, the ability to hear high-frequency sounds is affected first, followed by the ability to hear low-frequency sounds. The reason remains unknown. However, patients are more likely to report hearing difficulties even if only their ability to hear low-frequency sounds is affected, as this is the frequency of speech. 26

The treatment intensity of the photon or X-ray beam ranged from 8 to 18 megavoltage (MV), applied directly to the tumor, and decreased as it traveled to the tumor's periphery. Despite the efforts to avoid essential organs, such as the inner ear and brainstem, the amount of radiation reaching this undesirable area can be between 1.8 and 2.0 Gy for each exposure. 27 Theories that explain how radiation affects the inner ear are described as follows:

- Direct damage to the deoxyribonucleic acid (DNA) in the mitochondria of the inner hair cells and indirect damage from the formation of reactive oxygen species and reactive nitrogen species, resulting in DNA breakage. This occurs 1 hour after irradiation. 28
- The activation of proinflammatory cytokines produced by macrophages, such as tumor necrosis factor (TNF) α, IL-1, IL-6, and IL-8, causes mitotic arrest or leads to hair cell apoptosis via the p53-dependent or independent pathways. This occurs 6 hours after irradiation. 29
- Injury to the endothelial cells of the stria vascularis impairs the K+ recycling channel, rendering it unable to maintain the endolymphatic membrane potential. This also happens to the myelin nerve sheath and connective tissue cells of the auditory nerve., and it occurs 24 to 72 hours after irradiation. 30
- Both regenerative (epithelial resting and parenchymal cells) and non-regenerative cells (auditory hair and spiral ganglion cells) have lost some of their proliferative reverse capacity. This occurs 7 to 14 days after irradiation. 31

In our study, age > 50 years, T-stages 3 and 4, and radiation dose to the cochlea of > 43 Gy were associated with an increased risk of hearing loss in the univariate analysis, which was concordant with the results of previous studies. 22 23 According to our NTCP model, a maximum hearing loss risk was predicted to be 44.52% when both risk variables were present, and a minimum risk of 15.84% when neither risk factor was present. It is not surprising that a patient has some risk of hearing loss even with low-dose RT to the cochlea due to the well-documented dose–effect relationship for radiation damage to organized tissues. 32 Additionally, the aging process also had a major influence due to a decline in cellular repair capacity. 33

In our study, T-stages 3 and 4 and doses of radiation to the cochlea > 44 Gy were factors related to MEE. If both factors were present, the NTCP model indicated a maximum risk of MEE of 51.99%, whereas their absence was associated with a minimum risk of 20.42%. However, MEE was shown to have lower prevalence when compared with hearing loss due to its transient nature and tendency to fluctuate over time. Most of the patients had MEE prior to treatment because the disease itself obstructed the Eustachian tube. 34 During the follow-up period, incomplete physical examinations and medical records were found, resulting in the exclusion of a substantial number of patients with MEE from the study.

The present study has some limitations. First, it is possible that hearing loss detection during the follow-up period was underestimated. Prior to the establishment of the recommended guidelines in 2012, a follow-up audiogram was not indicated unless the patient reported hearing problems. Second, the result may not be possible to generalize to certain patient categories, such as stage-I patients who solely had definitive RT without concurrent chemotherapy, and patients with distant metastases. Third, our model was developed based on IMRT or VMAT patients; thus, its use in proton therapy patients might be limited. Future prospective multicenter studies are needed for external validation of the model and to increase the generalizability of the study's findings.

## Conclusion

The main risk factors associated with the occurrence of hearing loss and MEE were age, radiation dose to the cochlea, and tumor stage. Our model is useful in determining the risk factors, hence facilitating treatment decision-making.
